# AI-driven healthcare: a trend toward better healthcare or the emergence of public health burden

**DOI:** 10.3389/fdgth.2026.1845875

**Published:** 2026-05-13

**Authors:** Virak Sorn, Techly San, Sokchan Lorn

**Affiliations:** 1 Faculty of Health Sciences and Biotechnology, University of Puthisastra, Phnom Penh, Cambodia; 2 Faculty of Dentistry, University of Puthisastra, Phnom Penh, Cambodia

**Keywords:** AI in healthcare, algorithmic bias, artificial intelligence, clinical decision, ethics and regulation of AI

## Abstract

Recently, artificial intelligence (AI) has become a potent and innovative branch of computer science that is rapidly transforming healthcare delivery across the world. With its promise of improved diagnostics, individualized care, and effective health service management, it has the potential to fundamentally change medical practice and healthcare delivery. Yet, despite its potential, AI's growing influence also raises serious concerns about algorithmic bias, data misuse, over-reliance, and even the high risk of causing the emergence of a public health burden. This article critically examines recent breakthroughs in the application of AI in healthcare, whether AI represents a genuine advance toward better healthcare services or whether it could inadvertently contribute to public health risk. It is then argued that while AI's benefits are undeniable, its unregulated or poorly designed deployment could cause a growing public health burden. A balanced, human-centered, and ethical approach to AI adoption is therefore essential to ensure that digital progress translates into real health gains and discusses the possible future direction of AI-augmented healthcare systems.

## 1 Introduction

Artificial intelligence (AI) is increasingly embedded within the global health ecosystem, driving transformative innovations in areas such as diagnostics, clinical decision-making, drug discovery, and health system management (1, 2). Through advanced machine learning techniques, AI systems can analyze large and complex medical datasets to identify patterns and generate predictions with unprecedented speed and precision (3). In recognition of its expanding role, World Health Organization (WHO) published a discussion paper in 2024 examining the growing application of AI in the development and deployment of medicines, vaccines, and other health-related technologies, while also highlighting associated ethical concerns and governance challenges (4–6). Together, these developments underscore the considerable potential of AI to improve health outcomes and address previously unmet healthcare needs.

In many respects, the rapid advancement of AI represents a technological transformation comparable to landmark innovations such as antibiotics and vaccines, fundamentally reshaping how healthcare is delivered and how diseases are detected, managed, and understood. However, its accelerated integration into health systems also presents a critical paradox (7). While AI offers significant promise for improving clinical outcomes, enhancing efficiency, and expanding access to care, it may simultaneously introduce new risks and unintended consequences. Concerns have emerged that unchecked automation, algorithmic bias, and excessive reliance on data-driven systems could undermine clinical judgment, reinforce existing health inequities, and influence population health behaviors in undesirable ways (5–8). Moreover, the widespread use of digital technologies may contribute to emerging public health challenges, including technology-related conditions and broader psychosocial impacts.

Importantly, AI in healthcare is not a monolithic concept but rather encompasses a diverse range of approaches and tools with distinct purposes and implications. Broadly, AI applications can be categorized into generative AI and task-specific (clinical) AI. Generative AI—designed to produce content such as text, images, or clinical documentation—has demonstrated utility in areas including patient communication and medical education. However, it is also associated with notable limitations, including the risk of inaccurate or “hallucinated” outputs, inconsistency, and a lack of contextually grounded clinical reasoning (8, 9). In contrast, task-specific AI is developed for clearly defined functions such as diagnosis, prognosis, and triage and typically requires rigorous validation processes—including external validation and real-world performance evaluation—before safe integration into clinical practice (10). Distinguishing between these categories is essential for accurately assessing their respective benefits and risks and for ensuring that appropriate evaluation frameworks and regulatory standards are aligned with their intended use.

Against this backdrop, AI-driven healthcare raises an important question: does it represent a pathway toward better healthcare, or could it inadvertently contribute to a growing public health burden? Addressing this question requires a balanced examination of both the opportunities and risks associated with AI. Therefore, this article explores this duality while outlining key priorities for ensuring a safe, equitable, and ethically governed future for digital health.

## 2 Methods

This study utilized narrative review methodology to synthesize existing evidence on the opportunities, risks, and ethical implications of AI in healthcare. The review integrates literature on clinical applications (e.g., diagnostics, personalized care, and health system efficiency) with emerging evidence on methodological limitations, ethical concerns, and governance challenges, with a particular focus on implications for global and public health.

### 2.1 Literature search and source selection

A comprehensive literature search was conducted across multiple sources, including peer-reviewed journals, international policy reports, and grey literature. Key databases included Google Scholar, PubMed, Scopus, Web of Science, and IEEE Xplore, ensuring coverage of both clinical and technical perspectives on AI in healthcare. Additional materials were obtained from major international organizations, such as the World Health Organization, to incorporate up-to-date policy, ethical, and regulatory insights.

The review emphasizes studies published between 2019 and 2026 to reflect the most recent developments and applications of AI in healthcare. Search strategies employed combinations of relevant keywords, including “artificial intelligence,” “machine learning,” “digital health,” “clinical decision support,” “diagnostic accuracy,” “algorithmic bias,” “data privacy,” “ethical challenges,” and “AI governance,” to ensure a comprehensive and systematic identification of relevant literature.

### 2.2 Eligible criteria

Literature was selected based on relevance to the following key areas: (1) applications of AI in healthcare, including diagnostics, predictive analytics, personalized medicine, and health system management; (2) methodological challenges in AI development and evaluation, including overfitting, data leakage, shortcut learning, and generalizability; (3) clinical effectiveness and real-world implementation of AI systems; (4) ethical, legal, and social implications, including algorithmic bias, health inequities, data privacy, and cybersecurity risks; (5) health system impacts, including efficiency, access to care, and workforce implications; (6) governance frameworks, regulatory approaches, and strategies for responsible and human-centered AI.

## 3 AI as a catalyst for better healthcare services

### 3.1 Enhancing diagnostic accuracy

AI has demonstrated substantial potential to improve diagnostic accuracy across multiple areas of medicine. In particular, systems based on deep learning algorithms have shown strong performance in interpreting medical images and detecting conditions such as cancer, diabetic retinopathy, pneumonia, stroke, and Parkinson's disease (11–14). By analyzing large volumes of imaging data, these models can identify subtle pathological features that may not be readily detectable by the human eye, enabling earlier diagnosis and more timely intervention. Indeed, several validation studies report that AI-assisted radiology tools can achieve diagnostic performance comparable to that of experienced clinicians in specific clinical contexts (1, 11–16).

Beyond imaging, AI further enhances diagnostic processes through predictive analytics, laboratory data interpretation, and the analysis of electronic health records (EHRs) (8). By integrating diverse datasets, AI systems can uncover patterns associated with disease risk, allowing clinicians to identify conditions at earlier stages—even before clinical symptoms emerge (8–10). These capabilities support a shift toward more proactive and preventive models of care.

The application of AI-enabled diagnostic technologies is expanding across specialties such as radiology, pathology, dermatology, and ophthalmology. For example, AI-assisted analysis of retinal images has proven effective in detecting early signs of diabetic retinopathy through detailed assessment of retinal vasculature, thereby improving opportunities for timely treatment and better patient outcomes (3). As these technologies continue to evolve, their diagnostic accuracy and reliability are steadily improving, reinforcing their potential to enhance clinical decision-making and optimize patient care.

However, the performance of diagnostic AI systems is highly dependent on the quality and integrity of the underlying data. In particular, the reliability of labeled and annotated datasets plays a critical role in determining model accuracy. These datasets are typically derived from clinical interpretations, which may vary depending on the expertise of annotators, institutional practices, and diagnostic criteria. Inconsistent or noisy labels can introduce systematic errors during model training, undermining clinical validity (17). Consequently, increasing emphasis is placed on establishing expert-validated ground truth, standardizing annotation processes, and incorporating measures such as inter-observer agreement to ensure data integrity (18).

Equally important are robust validation strategies that extend beyond internal model testing. Best practices include external validation using independent datasets, prospective evaluation in real-world settings, and alignment with clinical workflows (19, 20). Without such measures, AI systems risk overfitting to development data or exploiting spurious correlations that do not generalize beyond the original training environment (21). Continuous performance monitoring following deployment is therefore essential to detect performance drift and maintain safety in clinical practice.

### 3.2 Enabling personalized and predictive care

AI offers significant opportunities to advance personalized and predictive healthcare, with broad implications for public and global health. Within modern healthcare systems, AI can support the development of more individualized care models by integrating diverse data sources, including genomic information, clinical histories, and lifestyle factors. Through the analysis of these complex datasets, AI enables the delivery of precision medicine tailored to the specific characteristics and needs of individual patients (14, 15).

Predictive algorithms can assist clinicians in forecasting disease progression, optimizing treatment strategies, and minimizing potential adverse effects. In oncology, AI-assisted systems have been used to support the selection of chemotherapy regimens based on patient-specific clinical and molecular profiles. Similarly, in the management of chronic diseases, predictive models can enhance monitoring, identify early warning signs of disease deterioration, and support preventive interventions (16). These capabilities allow healthcare providers to intervene earlier and design more targeted treatment strategies.

In neurology, AI-assisted technologies are also contributing to more personalized treatment approaches. For instance, advanced computational tools are increasingly used to support interventions such as deep brain stimulation (DBS), which plays an important role in the management of neurological conditions, including Parkinson's disease (22). By analyzing patient-specific clinical and neurological data, AI-driven models can help guide treatment planning and improve therapeutic outcomes.

Through predictive analytics, AI enables clinicians to detect diseases earlier, monitor disease progression more effectively, and tailor treatments according to individual patient needs (23). Collectively, these developments represent a shift from a predominantly reactive model of care toward a more proactive and preventive healthcare approach. Such a transformation has the potential to improve health outcomes, enhance patients' quality of life, and reduce healthcare costs by enabling earlier and more targeted interventions.

### 3.3 Increasing efficiency and access

AI has an increasingly important role in improving the efficiency of healthcare delivery and expanding access to services. Automated administrative systems can significantly reduce the burden of routine paperwork, allowing clinicians to devote more time to direct patient care. In addition, AI-driven telemedicine platforms facilitate healthcare delivery to remote and resource-limited settings, helping bridge gaps in the availability of medical expertise and services (24). Digital triage tools, chatbots, and clinical decision support systems can provide real-time guidance to both patients and healthcare professionals, thereby enhancing timely decision-making and promoting more equitable access to care (25).

Recent research has demonstrated the effectiveness of AI applications in disease diagnosis and clinical decision-making. As AI technologies continue to evolve, their diagnostic accuracy and operational efficiency are steadily improving (26). One of the key advantages of AI in healthcare lies in its capacity to enhance both the performance and availability of health service systems. Healthcare facilities globally face increasing pressure due to rising patient demand, shortages of healthcare workers, and expanding administrative responsibilities. AI-based systems can support health system operations by automating routine tasks and streamlining workflows, thereby improving overall system efficiency (27).

AI applications can automate several administrative functions, including patient scheduling, medical record management, clinical documentation, billing, and insurance processing. By reducing the time required for these activities, healthcare professionals can allocate more time to direct patient care, ultimately improving productivity, patient satisfaction, and service quality (27, 28). Furthermore, AI technologies can significantly expand access to healthcare services, particularly in underserved or geographically isolated communities. AI-enabled telemedicine solutions can facilitate remote consultations, triage services, and preliminary diagnostic support for patients who may otherwise face barriers to accessing healthcare due to distance, limited mobility, or shortages of healthcare providers (29).

Digital health technologies also enable patients to receive care from their homes through remote communication with healthcare professionals. In clinical decision support systems, AI assists clinicians by generating real-time recommendations based on established protocols and patient-specific data. Such systems are particularly valuable in low-resource settings where access to specialist expertise may be limited (30). By supporting evidence-based decision-making and improving the efficiency of healthcare operations, AI has the potential to enhance equity in healthcare access across different populations and regions. As AI technologies continue to advance, they are expected to further improve operational efficiency, reduce healthcare costs, and expand access to quality care, ultimately becoming an integral component of health systems worldwide (31).

## 4 Emerging risks and new threats to public health

Despite its promise, AI introduces risks that could paradoxically undermine public health and the global health context. These risks are not merely technical—they extend to behavioral, ethical, and social dimensions that may give rise to a public health burden. As discussed, below are the key factors that could contribute to a growing public health risk if the use of AI were not regulated and monitored properly.

### 4.1 Algorithmic bias and health inequities

AI models rely heavily on the quality and representativeness of the data used to train them. However, healthcare datasets often reflect historical and structural inequalities within health systems. When these datasets disproportionately represent certain ethnic, socioeconomic, or geographic populations, the resulting algorithms may produce inaccurate or discriminatory outcomes for underrepresented groups.

Evidence from prior research illustrates the magnitude of this challenge. According to a previous study by Obermeyer et al., an algorithm used to guide care management for millions of patients in the United States systematically underestimated disease risk among black patients. The algorithm relied on healthcare expenditure as a proxy for health need, leading it to incorrectly infer that “black patients” were healthier than equally ill “white patients” simply because less healthcare spending was recorded for them. This example highlights how biased data inputs can reinforce existing disparities in access to care and resource allocation (32).

Algorithmic bias is not limited to racial disparities but can also manifest across gender lines. In cardiovascular medicine, for instance, many predictive models have historically been developed using datasets dominated by male participants. Yet, cardiovascular disease often presents differently in women, and symptoms of heart attacks in women are frequently under-recognized or misdiagnosed. Consequently, algorithms trained primarily on male-centric datasets may fail to accurately identify risk patterns in female patients, potentially contributing to delayed diagnosis and suboptimal treatment outcomes (33, 34).

If left unaddressed, such biases may perpetuate structural inequalities in healthcare and undermine trust in emerging digital health technologies. These concerns are particularly relevant for low- and middle-income countries, where limited availability of diverse and representative health data may further constrain the equitable development and deployment of AI systems. Addressing algorithmic bias therefore requires deliberate efforts to ensure inclusive data collection, transparent model development, and robust regulatory oversight to promote fairness and equity in AI-driven healthcare.

### 4.2 Over-reliance on AI and loss of clinical judgment

While AI offers significant benefits for healthcare decision-making, its increasing integration into clinical practice raises concerns about over-reliance on algorithmic systems. One key issue is the phenomenon of “automation bias,” in which clinicians may place excessive trust in algorithm-generated recommendations without sufficiently questioning or critically evaluating the outputs. Such reliance can increase the risk of diagnostic errors or inappropriate treatment decisions, particularly when AI systems produce inaccurate or contextually inappropriate results (35).

Beyond immediate clinical risks, sustained dependence on AI tools may gradually weaken clinicians' diagnostic reasoning and professional judgment. Clinical decision-making traditionally relies on a combination of scientific knowledge, clinical experience, and patient-centered evaluation. However, when AI systems routinely guide diagnostic or therapeutic decisions, healthcare professionals may become less inclined to engage in independent analytical thinking. In extreme cases, clinicians risk becoming passive operators of algorithmic tools rather than active decision-makers, potentially diminishing professional autonomy and undermining the humanistic elements of medical care (36).

Another emerging concern is that AI-driven healthcare could inadvertently contribute to new forms of health risk. Excessive dependence on digital tools and automated systems may foster technology reliance among both clinicians and patients. Additionally, inaccurate algorithmic analysis or poorly validated models may lead to misdiagnosis, inappropriate treatment pathways, or delayed care. These scenarios illustrate how AI systems, while designed to enhance healthcare delivery, may also introduce new types of clinical and systemic harm that require careful oversight and regulation (37).

Taken together, these concerns highlight a broader ethical and professional challenge in modern healthcare: ensuring that AI functions as a supportive clinical tool rather than a replacement for human judgment. Maintaining a balanced relationship between technological innovation and clinical expertise will be essential to safeguard patient safety, preserve the integrity of medical practice, and prevent unintended health burdens associated with technological over-dependence.

### 4.3 Data privacy and cybersecurity threats

The rapid integration of AI into healthcare systems has intensified concerns regarding data privacy and cybersecurity. Healthcare institutions manage vast amounts of highly sensitive personal information, including medical histories, genetic data, and biometric records. As digital health platforms, AI models, and cloud-based infrastructures become increasingly interconnected, the risk of cyberattacks, data breaches, and unauthorized data access correspondingly increases. Such vulnerabilities can expose confidential patient information, potentially leading to identity theft, financial fraud, insurance discrimination, and psychological distress among affected individuals (38).

Beyond direct security threats, the use of large-scale health datasets to train AI models also raises complex ethical and legal questions. In many cases, personal health data may be reused for algorithm development without fully transparent consent processes or clear governance frameworks. This situation raises concerns related to data ownership, patient autonomy, and the potential for surveillance or misuse of sensitive health information (39, 40). Without robust regulatory safeguards and strong cybersecurity measures, the expansion of AI-driven healthcare systems could undermine public trust and compromise patient confidentiality. Therefore, ensuring secure data management, transparent consent mechanisms, and effective regulatory oversight is essential for protecting patient rights and maintaining ethical standards in the evolving digital health landscape.

## 5 Ethical and regulatory challenges

The growing integration of AI into healthcare also raises significant ethical and regulatory challenges, particularly in relation to accountability, harm prevention, and the protection of vulnerable populations. Ethical concerns arise when AI systems are designed or optimized in ways that prioritize user engagement or efficiency without fully considering potential psychological or behavioral consequences. In some cases, such systems may inadvertently contribute to dependency, cognitive overload, or mental health harms, while the responsibility for such outcomes remains unclear or diffused across multiple actors involved in the development and deployment of the technology (41).

Issues of informed consent, transparency, and autonomy are also central to the ethical debate surrounding AI in healthcare. Many users may not fully understand how AI systems collect, process, and interpret their data or how algorithmic recommendations may influence their health-related decisions and behaviors. These concerns are particularly relevant for vulnerable groups, including children and adolescents, who may have limited capacity to critically evaluate digital technologies and their potential impacts (42). Without clear communication and transparent algorithmic processes, the use of AI in healthcare risks undermining patient autonomy and trust.

From a regulatory perspective, existing legal and governance frameworks often struggle to keep pace with the rapid evolution of AI technologies. Current health and technology regulations frequently lack clear provisions for addressing AI-related psychological or behavioral harms, resulting in gaps in oversight and accountability. Regulators also face challenges in defining measurable forms of harm, establishing appropriate safety standards, and determining liability among multiple stakeholders, including developers, healthcare providers, technology companies, and platform operators (41).

Recognizing these challenges, WHO has emphasized the importance of establishing comprehensive governance frameworks to ensure that AI systems are safe, ethical, and aligned with human well-being. Such frameworks should incorporate mechanisms for risk assessment, continuous monitoring, transparency, and accountability in the development and deployment of AI technologies (43). Nevertheless, the global governance landscape for AI in healthcare remains fragmented and insufficiently developed. Without harmonized standards, robust regulatory oversight, and ethical safeguards, the potential for AI technologies to cause unintended harm—whether through design flaws, misuse, or inadequate governance—remains a significant concern.

In summary, recent research consistently demonstrates that AI does not cause the emergence of new illnesses. Instead, it introduces a network of iatrogenic, behavioral, and system-level risks that arise from interactions between humans, algorithms, and healthcare systems. This represents a paradigm shift from viewing AI risks as isolated model errors to understanding them as systemic vulnerabilities embedded across clinical workflows and healthcare infrastructures.

## 6 Limitations in AI evaluation and methodological challenges in healthcare AI

Recent studies suggest that the evaluation of AI models in healthcare should extend beyond conventional performance metrics such as accuracy or area under the curve (AUC), as these measures alone may not fully capture clinical reliability or ensure safe real-world deployment (44, 45). Contemporary research highlights several methodological challenges that can affect the validity, robustness, and reproducibility of AI systems in medical contexts.

### 6.1 Overfitting in medical AI

Overfitting remains a persistent concern in healthcare machine learning, particularly in applications involving medical imaging and relatively small or homogeneous clinical datasets. It arises when models learn patterns that are highly specific to the training data, including noise or dataset-specific artifacts, rather than underlying clinical signals that generalize across populations. While such models may demonstrate strong performance during internal validation, their effectiveness often declines when applied to external datasets or real-world settings. This issue underscores the importance of incorporating external validation, diverse datasets, and appropriate regularization techniques in the development of clinical AI systems (46).

### 6.2 Shortcut learning and spurious correlations

Shortcut learning has increasingly been recognized as a potential limitation in medical AI. Instead of identifying clinically meaningful features, models may rely on spurious correlations, such as imaging artifacts, differences in acquisition devices, or site-specific characteristics. Although these shortcuts can lead to high apparent performance during testing, they may reduce model robustness when deployed in new clinical environments. Emerging evidence suggests that deep learning models can be particularly susceptible to such behavior, raising important considerations for reliability and fairness in clinical decision support applications (47, 48).

### 6.3 Data leakage and reproducibility issues

Data leakage represents a critical methodological issue in AI research, occurring when information from validation or test datasets is inadvertently incorporated into the training process. This may result from improper dataset splitting, preprocessing prior to partitioning, or overlap of patient samples across datasets. Such practices can lead to overly optimistic performance estimates and limit the reproducibility of findings. Addressing data leakage requires careful experimental design, transparent reporting, and the use of rigorous validation strategies, including appropriate cross-validation techniques (49).

### 6.4 Limited generalization across clinical settings

Another important challenge is the limited generalizability of AI models across different clinical settings. Models trained on narrowly defined datasets may not perform consistently when applied to populations with different demographic characteristics, disease prevalence, or clinical practices. Variations in imaging protocols and healthcare infrastructure can further contribute to performance variability. Studies have reported that model performance often declines during external validation, highlighting the need for multi-center datasets and ongoing evaluation following deployment (50).

### 6.5 Implications for clinical reliability

Taken together, these methodological considerations—including overfitting, shortcut learning, data leakage, and limited generalization—highlight key challenges in the development and evaluation of AI systems for healthcare. If not adequately addressed, they may affect model reliability and limit safe clinical implementation. Accordingly, continued efforts to strengthen validation frameworks, improve transparency in reporting, and incorporate diverse datasets are likely to play an important role in enhancing the robustness and trustworthiness of AI in clinical practice (45).

## 7 Toward responsible and human-centered AI in healthcare

Ensuring that AI contributes positively to healthcare requires a deliberate shift toward responsible and human-centered implementation. Several strategic actions are essential to ensure that AI systems enhance healthcare delivery while safeguarding ethical principles, human values, and patient well-being.

- First, human oversight must remain central to AI-enabled healthcare systems. AI technologies should be designed to support—rather than replace—clinical expertise and professional judgment, ensuring that healthcare providers retain ultimate responsibility for medical decision-making (44, 51).
- Second, fairness and inclusivity must be prioritized in the development and deployment of AI systems. To minimize the risk of algorithmic bias and protect marginalized populations from potential harm, AI models should be trained using diverse and representative datasets. Regular auditing and validation of algorithms are also necessary to detect and correct biases that may emerge during implementation (52). These measures are particularly important for ensuring equitable healthcare outcomes across different demographic and socioeconomic groups (53).
- Third, transparency and explainability are critical components of trustworthy AI systems. Clinicians and patients should be able to understand how AI-generated recommendations are produced and how they influence clinical decisions. Transparent algorithmic processes not only enhance interpretability but also foster trust and acceptance among healthcare professionals and the public (54).
- Fourth, strong data governance frameworks and privacy protections are essential to safeguard sensitive health information. Robust cybersecurity measures, clear consent mechanisms, and responsible data stewardship practices are necessary to maintain confidentiality and public confidence in AI-driven healthcare technologies (54–56).
- Finally, adaptive regulatory frameworks and continuous monitoring mechanisms are required to evaluate the long-term impacts of AI systems, identify unintended consequences, and respond to emerging risks (56–60).

Collectively, these measures support the development of an AI ecosystem in healthcare that is ethical, accountable, and aligned with human-centered principles. By prioritizing safety, equity, and transparency, healthcare systems can harness the benefits of AI while minimizing potential harms, ensuring that technological innovation ultimately serves the needs and well-being of patients and communities. As summarized in Table 1, AI-driven healthcare offers substantial gains in efficiency while also presenting significant ethical and clinical challenges, underscoring the importance of human-centered implementation. This illustrates the dual nature of AI in healthcare, highlighting both its potential to improve patient outcomes and the risks that demand careful ethical consideration and robust regulatory oversight.

**Table 1:** Summary of applications of AI in healthcare: benefits, risks, and mitigations.

AI application category	Specific use case	Clinical & reported benefits	Potential risks, harm & ethical concerns	Mitigations
Precision (Medicine/Predictive Care)	AI integrates genomic data, clinical histories, and lifestyle information for individual patients. It is also used for Electronic Health Record (EHR) mining to identify patients at risk before symptoms manifest.	Tailored chemotherapy selection; proactive monitoring and prevention; reduced healthcare costs; optimized treatment strategies; shifting from reactive to proactive care; improved quality of life.	Algorithmic bias; perpetuating structural inequities; data privacy and cybersecurity threats; lack of accountability for damage; inaccurate analysis leading to new forms of harm.	Use diverse and high-quality datasets to minimize bias; validate models across populations; implement continuous monitoring to maintain performance across demographic groups.
Diagnostic (Imaging/Diagnostics)	Interpreting medical images for cancer, diabetic retinopathy, pneumonia, stroke, and Parkinson's disease.	High diagnostic accuracy on par with skilled clinicians; detection of subtle pathological features invisible to humans; earlier intervention and improved patient prognosis.	Risks include automation bias (over-trusting outputs without critical evaluation), erosion of clinical reasoning skills, and the loss of professional autonomy.	Adopt explainable AI methods; require rigorous clinical validation and external benchmarking; maintain clinician oversight to verify AI-generated outputs.
Healthcare (Delivery, Access & Admin)	Telemedicine platforms, digital triage tools, chatbots, automated administrative systems, and decision support systems.	Extending care to remote or resource-limited areas; reducing administrative paperwork; providing real-time assistance; bridging medical expertise gaps.	Data privacy breaches; cybersecurity threats; dehumanization of medicine; unchecked automation; risk of maximizing engagement leading to dependency.	Establish regulatory and ethical frameworks; ensure data privacy and cybersecurity; integrate AI into workflows with human-in-the-loop approaches and workforce training.

## 8 Conclusions

AI-driven healthcare has the potential to revolutionize medicine by improving accuracy, efficiency, and accessibility. However, without ethical safeguards and robust governance, AI could also contribute to new forms of health-related harm through misdiagnosis, psychological stress, inequity, and loss of human connection. The challenge, therefore, is not whether AI should be used in healthcare, but how it should be used. AI's success implemented in healthcare depends on maintaining the right balance between innovation and human oversight. A future where AI truly enhances human health will require transparency, inclusivity, and strong ethical foundations. In this evolving landscape, technology must remain a tool to heal—not a cause of harm.

## Data Availability

The original contributions presented in the study are included in the article/Supplementary Material, further inquiries can be directed to the corresponding author.
